# XPR1: a regulator of cellular phosphate homeostasis rather than a Pi exporter

**DOI:** 10.1007/s00424-024-02941-0

**Published:** 2024-03-20

**Authors:** David Burns, Rolando Berlinguer-Palmini, Andreas Werner

**Affiliations:** 1 Biosciences Institute, Newcastle University, Framlington Place, Newcastle Upon Tyne, NE2 4HH UK; 2 Bio-Imaging Unit, The Medical School, Newcastle University, Newcastle Upon Tyne, NE2 4HH UK

**Keywords:** XPR1, Phosphate exporter, *Xenopus* oocytes, Cellular phosphate balance

## Abstract

Phosphate (Pi) is an essential nutrient, and its plasma levels are under tight hormonal control. Uphill transport of Pi into cells is mediated by the two Na-dependent Pi transporter families SLC34 and SLC20. The molecular identity of a potential Pi export pathway is controversial, though XPR1 has recently been suggested by Giovannini and coworkers to mediate Pi export. We expressed XPR1 in *Xenopus* oocytes to determine its functional characteristics. *Xenopus* isoforms of proteins were used to avoid species incompatibility. Protein tagging confirmed the localization of XPR1 at the plasma membrane. Efflux experiments, however, failed to detect translocation of Pi attributable to XPR1. We tested various counter ions and export medium compositions (pH, plasma) as well as potential protein co-factors that could stimulate the activity of XPR1, though without success. Expression of truncated XPR1 constructs and individual domains of XPR1 (SPX, transmembrane core, C-terminus) demonstrated downregulation of the uptake of Pi mediated by the C-terminal domain of XPR1. Tethering the C-terminus to the transmembrane core changed the kinetics of the inhibition and the presence of the SPX domain blunted the inhibitory effect. Our observations suggest a regulatory role of XPR1 in cellular Pi handling rather than a function as Pi exporter. Accordingly, XPR1 senses intracellular Pi levels via its SPX domain and downregulates cellular Pi uptake via the C-terminal domain. The molecular identity of a potential Pi export protein remains therefore elusive.

**Supplementary Information:**

The online version contains supplementary material available at 10.1007/s00424-024-02941-0.

## Introduction

Inorganic phosphate (Pi) is essential to life, and its homeostasis is controlled tightly in all organisms. Synthesis of biomolecules as well as energy metabolism and signalling crucially rely on adequate levels of Pi. Human plasma phosphate levels depend on intestinal uptake and bone remodelling and are maintained at around 1 mM by controlled renal excretion [19].

Dietary Pi is taken up in the small intestine predominantly through passive, paracellular movement along its electrochemical gradient. Only under conditions of Pi starvation energy-dependent transcellular uptake via the Na-phosphate cotransporter SLC34A2 becomes physiologically important [12, 13, 21, 22].

Bones store much of total body Pi which can be mobilized in states of low Pi and Ca^2+^ levels, crucially regulated by parathyroid hormone (PTH) and—indirectly—by 1,25(OH)_2_ vitamin D_3_. Endocrine signalling also affects transcellular Pi absorption in the intestine and controls renal excretion to maintain body Pi homeostasis [16, 17]. In the kidney, Pi is freely filtered in the glomerulus followed by transcellular re-uptake from the primary urine in the proximal tubule. The energy-dependent transport across the apical brush border membrane is mediated by the two Na-dependent transporters SLC34A1 and SLC34A3. SLC34A1 displays a 1Pi/3Na stoichiometry whereas SLC34A3 transports 1Pi with 2Na. Urinary Pi excretion is under tight control of PTH and fibroblast growth factor 23 (FGF-23) which both inhibit Pi re-uptake via downregulation of SLC34A1 and SLC34A3 [12]. The proximal tubular cells in the kidney also play a defining role in sensing and signalling whole-body plasma Pi levels. Accordingly, phosphate uptake stimulates glycolysis and the production of the metabolite glycerol-3-phosphate (G3P). G3P is released into the circulation and triggers the synthesis and release of FGF23, which in turn causes phosphaturia and closes the feedback loop [32].

The endocrine regulation of Pi homeostasis includes primarily the mentioned intestine, bones and kidney, though every cell is in need of Pi for structural, metabolic and signalling purposes. Pi uptake in these cells is mediated by the ubiquitously expressed Na-dependent Pi transporters SLC20A1 and SLC20A2. Several lines of evidence including knockout mice indicate that SLC20A1 in particular is essential for normal muscle development and function [3, 7] and also drives cancer progression [24]. Of note, SLC20A1 and SLC20A2 have also been linked to extracellular Pi sensing in a process that involves Pi binding to the transporter rather than the transport and the concomitant increase in intracellular Pi [5].

The process of how Pi leaves cells after intracellular accumulation by SLC34 or SLC20 transporters is not well understood and controversial. XPR1 has emerged as the most promising candidate to mediate basolateral efflux of Pi from epithelia and other cells. Because of sequence similarities with plant and fungal proteins involved in phosphate metabolism, the protein was investigated in this context and found to stimulate the efflux of radioactive Pi [10, 28]. XPR1 is phylogenetically well conserved, and homologues are found in metazoans. XPR1 is broadly expressed in cell lines and various organs, also in tissues that are not primarily associated with Pi homeostasis [8, 10]. Mutations in XPR1 have been found in a cohort of patients with familial brain calcification, a condition that is also associated with mutations in phosphate transporters of the SLC20 family [8, 18]. Moreover, the kidney-specific conditional knock-out of XPR1 caused an increased urinary excretion of Pi. Concomitantly, these animals develop hypophosphatemic rickets and severe renal tubular dysfunction (Fanconi syndrome) [1]. Experiments in zebrafish have established an additional link between Pi homeostasis and XPR1 expression: XPR1 is required for osteoclast development, and mutants show impaired bone remodelling [23]. These findings lead to the claim that XPR1 was the main exit path for Pi from epithelia and other cell types [15]. The nature of the different phenotypes reported for XPR1 mutations suggests that cells with high transcellular Pi flux suffer from an impaired exit pathway leading to severe complications and even cell death [11]. Whereas the consequences of an XPR1 knockdown are well described, the functional properties of the protein—apart from stimulating Pi-flux, are rather unclear.

XPR1 function has been difficult to assess due to the lack of an efficient system for exogenous protein expression. The data presented in key papers are from endogenous XPR1 (in combination with siRNA knockdown controls) and from Pi export experiments with osteoclast-like cells (differentiated RAW264.7 cells), though the latter experiments did not specifically focus on XPR1 [10, 14]. Importantly, in both systems, efflux was stimulated by extracellular Pi indicating a bi-directional transport mode. Sulphate was excluded as a substrate for XPR1 while Pi analogues such as arsenate, phosphonoformic acid or pyrophosphate stimulated Pi efflux. Moreover, efflux was maximal under acidic conditions [10, 14]. A better understanding of XPR1 function would be commendable considering the possibility of modulating systemic Pi levels by reducing its efflux from renal tubular cells.

## Materials and methods

### Solutions

Modified Barth’s solution (MBS): NaCl, 88 mM; KCl, 1 mM; CaCl_2_, 0.4 mM; Ca(NO_3_)_2_, 0.33 mM; MgSO_4_, 0.8 mM; TRIS–HCl, 5 mM (pH 7.4); NaHCO_3_, 2.4 mM. Ca^2+^-free MBS, same as above but without CaCl_2_. Normal frog Ringer’s solution (NFR): NaCl, 90 mM; KCl, 2 mM; CaCl_2_, 2 mM; MgCl_2_, 1 mM; HEPES, 5 mM (pH 7.4).

### Xenopus oocytes

Excised ovarian sacs of *Xenopus* were purchased from the European *Xenopus* Resource Centre (Plymouth, UK). Oocytes were defolliculated using Collagenase A (Roche; 2 mg/ml in Ca^2+^-free MBS) followed by extensive washes with first Ca^2+^-free MBS and then MBS. Stage VI cells were sorted followed by injection the next day [20, 29]. Ten nanograms of in vitro transcribed RNA in 40 nl of water were routinely injected per oocyte. Injected cells were incubated at 18 °C for 3–7 days followed by Pi uptake and efflux measurements.

### Transport assays

The uptake of Pi and arginine (Arg) was performed as described [23] in NFR containing 0.2 mM Pi and ~ 30 µCi/ml ^33^P (Hartmann Analytics, Germany) as a tracer or 50 µM of Arg plus 5 µCi/ml ^3^H- Arg. For efflux measurements, oocytes were washed with NFR after an uptake of 20–30 min and distributed into single wells in 96 well plates. Efflux medium was added as specified in the “Results” section. Efflux was performed for 30 min. The efflux medium was removed, and radioactivity was counted using a Beckmann scintillation counter. In parallel, the corresponding oocytes were dissolved in 1% SDS and counted. Efflux was determined in % as follows:

$${{\text{dpm}}}_{{\text{efflux}}} / ({{\text{dpm}}}_{{\text{efflux}}} + {{\text{dpm}}}_{{\text{oocytes}}}) \times 100$$

Each construct and each condition were tested with at least three different batches of oocytes; 5–8 oocytes were tested for the specific conditions. If uptake was measured, data are presented as bar graphs with standard deviation, including one example of comparable experiments as oocytes from different frogs have different intrinsic transport activities for Pi. Efflux experiments are represented as box plots combining data from several batches of oocytes (the % calculation normalizes intrinsic transport). The centre lines show the medians, the crosses represent the sample mean. The box indicates the 25th and 75th percentiles as determined by R software; whiskers extend 1.5 times the interquartile range from the 25th and 75th percentiles.

### Constructs

To avoid the cloning of numerous cDNAs, PCR fragments of the relevant cDNAs were generated including a T7 RNA polymerase recognition motif at the 5′ end and a short polyA tail at the 3′ end. In brief, specific primers binding in the 5′ and 3′ untranslated regions of *Xpr1* were designed and used to amplify cDNA from *Xenopus* kidney total RNA. In a second PCR, tagged nested primers were used to add a T7 motif and a polyA tail of up to 20 residues. Overlapping PCR was used to either generate long template DNA (usually > 2500 base pairs) or attach fluorescent protein tags (Supplementary Fig. 1). All in vitro transcribed RNA was synthesized from PCR-generated fragments using the mMESSAGE mMACHINE™ T7 Transcription Kit (Thermo Fisher Scientific) including G(5′)ppp(5′)G RNA Cap Structure Analogue (NEB). In the first step, the cDNA sequences of candidate genes (*Xpr1*, *Kirrel*, *Myorg*, *Ip6k1/2*, *Ppip5k1/2* and *Kidins220* as well as *Slc26a1*, *Slc29a3*, *Slc37a3* and *Slc37a4*) were retrieved from Xenbase (https://www.xenbase.org/xenbase/) and primers were designed in the 5′ and 3′ untranslated regions and used to amplify the relevant DNA fragments by RT-PCR (Omniscript, Qiagen). Long cDNAs such as *Kidins220* [6] were amplified in two pieces and joined by overlapping PCR using GoTaq® Long PCR Master Mix (Promega). In the next step, nested primers with a 5′ T7 promoter and a 3′ poly A sequence, respectively, were used to amplify the template for in vitro transcription. The cDNA was purified and sequenced, and 0.2–0.5 µg were in vitro transcribed. To generate XPR1 fragments, a comparable strategy was applied with T7 promoter-tagged forward primers and polyA-tagged reverse primers. They bound within the XPR1 cDNA sequence at regions in between SPX and core domain as well as between the core domain and the C-terminus; the primers were used in combinations to generate individual domains of XPR1 (SPX-, core- and C-terminal domain). All primer sequences are listed in the supplementary material.

### Confocal microscopy

Red and green fluorescent protein tags were added to *Xenopus Xpr1* cDNA at either the N- or the C- terminus by overlapping PCR. Oocytes were assayed in glass bottom petri dishes using × 10 magnification and a Nikon A1R confocal microscope. Fluorescence was quantified using Image Fiji software.

## Results

*Xenopus* oocytes have been the expression system of choice to characterize transport properties of Pi transporters (SLC34) and other membrane proteins alike. Protein expression levels are particularly high and easily detectable per flux measurement or electrophysiology with Pi transporters from fish, i.e. flounder or zebrafish [9]. SLC34 transporters from humans and other mammals still express but with reduced efficiency, indicating a minor species barrier. Since XPR1 was identified as a Pi export transporter, several labs have attempted to express the protein in *Xenopus* oocytes to characterize its functional properties, though without success [15]. To avoid species incompatibility, we aimed to characterize the Pi efflux from frog oocytes using the *Xenopus laevis* isoform of XPR1. To avoid cloning of the relevant cDNAs, we pursued a PCR-based approach to generate in vitro transcribed RNA for expression purposes. To increase the intensity of a potential efflux signal, Slc34 from flounder (*fl*Slc34) [30] was co-expressed, and *Xenopus* oocytes were loaded with ^33^P prior to efflux measurements.

**Fig. 1:**
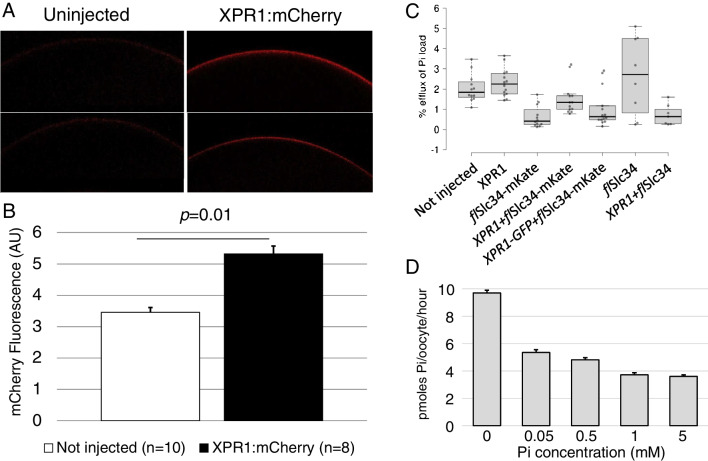
XPR1 is expressed at the membrane of *Xenopus* oocytes. Various constructs including the flurescent tags mCherry, mKate and GFP were tested with qualitatively comparable results for both localization and efflux of Pi. **A** mCherry-tagged XPR1 displays enhanced fluorescence (right panel) as compared to non-injected oocytes (left panel). **B** Quantification of fluorescence in arbitrary units. **C** *Xenopus* XPR1 wild type and tagged with fluorescent proteins did not affect Pi efflux from oocytes. **D** Native oocytes were exposed to varying concentrations of Pi for 24 h followed by measurement of Pi uptake. The reduction of Pi transport to even small increases in Pi in the medium indicates that oocytes have an endogenous system for sensing and responding to environmental Pi

In the first set of experiments, membrane localization of exogenous XPR1 was confirmed using constructs with either the 5′ or the 3′ end tagged with green or red fluorescent protein. Red fluorescence gave clearer results because of autofluorescence of yolk protein in the GFP spectrum. The XPR protein was clearly found at the membrane, with enhanced staining as compared to non-tagged XPR1 (background), indistinguishable from the pattern observed with RFP-tagged flounder Slc34 (Fig. 1A and B). Oocytes injected with *Xpr1*, *flSlc34* and a combination of both were then assessed by flux measurement for Pi uptake and efflux. Data are presented as a percentage of efflux compared to the total Pi uptake. These experiments revealed that under standard conditions, efflux into frog Ringer solution (pH 7.4, no Pi added) was minimal and not enhanced by XPR1. Moreover, tags did not affect Pi transport in either direction. In some experiments, XPR1 expression slightly reduced the uptake of Pi into non-injected oocytes—the effect is likely due to exogenous and endogenous membrane proteins competing for membrane localization and is not specific for Pi transport (Fig. 1C). Moreover, we exposed uninjected oocytes to increasing levels of Pi in the medium for 24 h and saw a concentration-dependent decrease in Pi uptake (Fig. 1D). The significant effect confirmed that oocytes express intrinsic components to sense Pi and adjust its uptake (Supplementary Fig. S2).

**Fig. 2:**
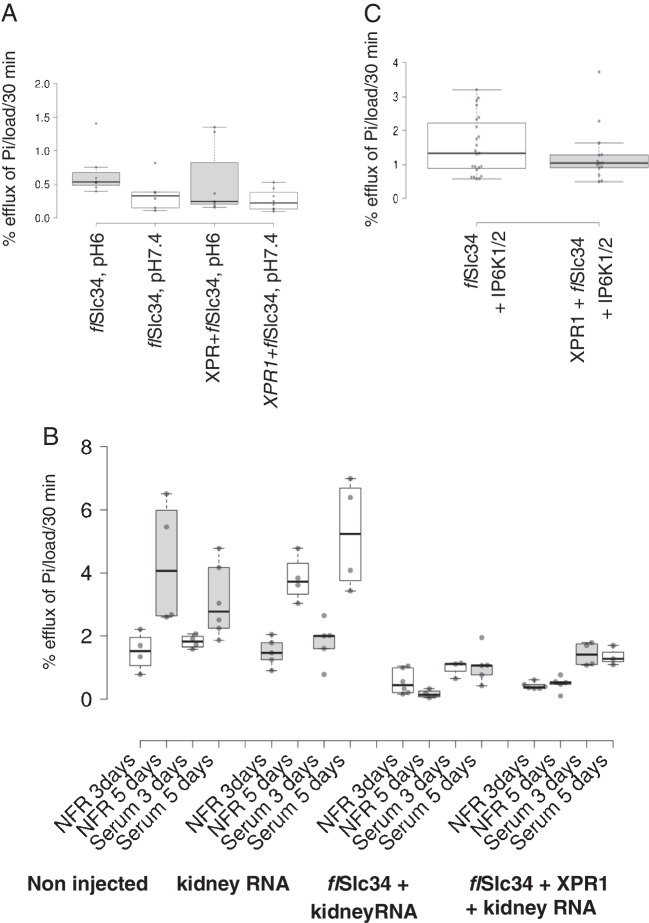
Pi efflux from oocytes injected with *XPR1*, *fl*Slc34 and potential cofactors under various experimental conditions. **A** Oocytes were injected with XPR1 and *fl*Slc34 + Xpr1 and efflux was assayed at pH 6 and 7.4, respectively. **B** Oocytes were injected with RNAs encoding XPR1, *fl*Slc34 and total kidney RNA from *Xenopus*. In general, oocytes with higher net uptake of Pi (i.e. expressing *fl*Slc34) tend to show lower efflux, as observed in many experiments. No significant effect of XPR1 on Pi export is detectable (compare the two groups on the right). The apparent change in efflux between 3 and 5 day incubation in groups 1 and 2 on the left reflects intrinsic changes in oocytes rather than the effects of injected material. A representative experiment is shown. **C** Oocytes were injected with *fl*Slc34 and a mix of pooled *IP6K1* and *IP6K2* cRNAs. Co-expression of the kinases did not affect Pi efflux. Oocytes from three different experiments are included

We hypothesized that the composition of the efflux medium could reduce or inhibit XPR1-mediated Pi transport. Early investigations, for example, suggested an exchange of Pi to occur at the basolateral membrane [14]. Hence, we tested various efflux conditions, including pH (pH 6 and pH 7.4, Fig. 2A), the addition of Pi to the export solution as well as using (goat) serum (Fig. 2B). All of these manoeuvres showed only minor effects with a slight but non-significant stimulation of pH 6 vs pH 7.4 and the addition of 1 mM Pi. Serum showed potentially the highest efflux, though the oocytes became very fragile (likely due to serum proteases) and potentially unreliable (Fig. 2B). Hence, future experiments were done with normal frog Ringer’s solution (NFR) pH 6 containing 1 or 2 mM Pi.

The failure of XPR1 to stimulate Pi export could be due to the lack of co-factors or inhibitory signals within the oocytes. To broadly test these possibilities, 50 ng of total RNA from *Xenopus* kidney was co-injected with combinations of *flSlc34* and *XPR1* and transport was measured after 3 and 5 days. Whereas efflux appeared to increase slightly between day 3 and day 5 in non-injected and kidney RNA-injected oocytes, no such effect could be assigned to XPR1. We also generated constructs for the two kinases IP6K1 and IP6K2 which produce the highly phosphorylated inositols that bind to XPR1. However, co-injection of cRNA for the two kinases did not stimulate Pi efflux in the presence of XPR1 (Fig. 2C). cRNA of other potential XPR1 modulators such as Kirrel, Myorg, IP6K1/2, PPIP5K1/2 and KIDINS220 were also co-expressed and again, failed to enhance Pi export in the presence of XPR1. Mutations in Kirrel and Myorg are, as XPR1, associated with familial brain calcification whereas IP6K1/2 and PPIP5K1/2 are additional kinases that phosphorylate IP5 and IP7, respectively [8]. KIDINS220 has recently been identified as an essential co-factor for XPR1 localization and function [6]. None of these interventions resulted in enhanced Pi efflux that could be associated with XPR1 (Supplementary Fig. 3).

**Fig. 3:**
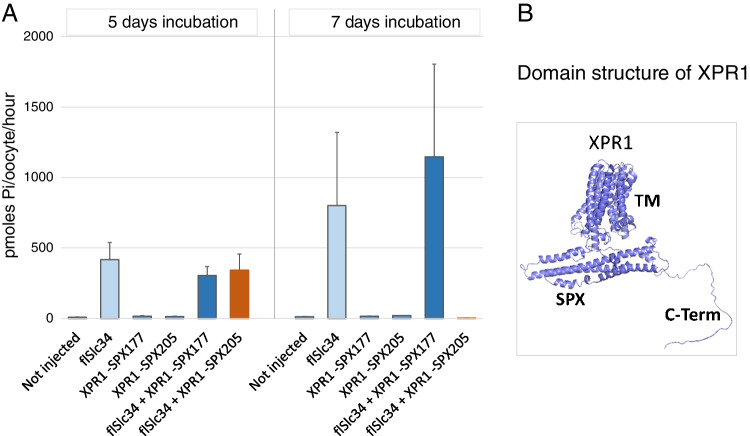
Pi uptake into oocytes expressing XPR1 constructs that lack the SPX domain. Constructs that lacked either part or the entire SPX domain were generated and expressed in oocytes. Oocytes were incubated for 5 days (left group) and 7 days (right group). Non-injected oocytes and the truncated constructs alone did not stimulate Pi uptake. *fl*Slc34 and *fl*Slc34 + XPR1-177 showed significant Pi uptake that increased and became more variable between days 5 and 7 (light and dark blue bars). *fl*Slc34 + XPR1-205 showed a downregulation of Pi uptake to basal level between day 5 and day 7 (orange bars). Right panel, alpha fold-based model of human XPR1. The individual protein domains are indicated: SPX, SPX-domain; TM, transmembrane domain or ‘core’ and C-Term, C-terminal domain

**Fig. 4:**
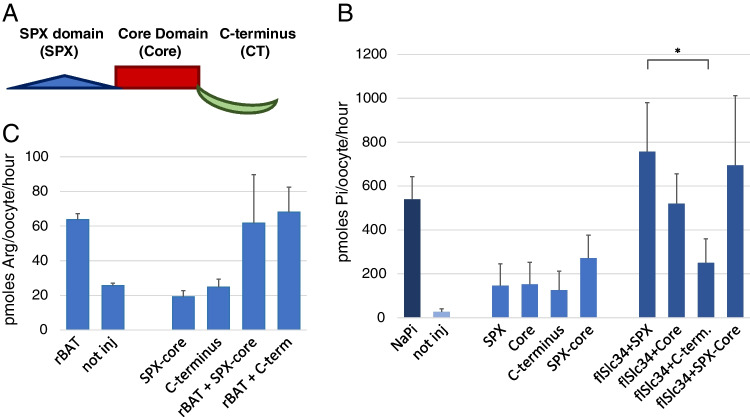
Pi uptake into oocytes expressing XPR1 protein domains. **A** Schematic representation of XPR1 with the domains SPX, core and C-terminus. **B** Cells were injected with constructs of XPR1 domains; SPX domain, core domain, the C- terminus and XPR1 without C-terminus (SPX-core) and assayed after 5 days. **C** The effect is specific for Pi transport as expression of XPR1 fragments has no effect on.^3^H arginine uptake into oocytes (representative experiment of 3 repetitions is shown). One-way ANOVA with Tukey’s post hoc test, *p < 0.05

A final avenue to rescue potential XPR1 Pi-export activity was to generate XPR1 mutants that lacked the SPX-signalling domain. Because Barth’s medium does not contain Pi, intracellular Pi in oocytes may be low, and the SPX domain could inhibit efflux activity. Moreover, the SPX domain was reported to be dispensable for XPR-mediated Pi efflux [10]. The structural model of XPR1 shows in essence three different domains, the N-terminal SPX domain comprising about 230 amino acids (aa), the transmembrane core with 8 TM helices (~ 390 aa) and the C-terminus (~ 90 aa, Fig. 3 right panel and Fig. 4A). Measurement of both uptake and efflux revealed minimal Pi efflux in oocytes expressing the truncated XPR1 constructs without SPX domain, comparable to background (not shown). Remarkably, however, the construct with the fully truncated SPX domain (XPR1-SPX205) had initially no effect on *fl*Slc34-mediated Pi uptake (5-day incubation, Fig. 3) but completely downregulated *fl*Slc34 upon long incubation (7-day incubation, Fig. 3). We interpreted this finding that the truncated XPR construct may be slowly expressed and/or diffuse slowly within the membrane to reach its target *fl*Slc34.

To test whether XPR1 was organized in functional modules, we generated constructs that encoded the individual domains (N-terminal SPX domain, core, C-terminus as well as XPR1 without C-terminus (Fig. 4A). The constructs were expressed in oocytes, either individually or together with *fl*Slc34 and assayed for Pi uptake after 2 (not shown) and 5 days (Fig. 4B). In order to dampen the variable intrinsic Pi uptake, one batch of oocytes was incubated for 30 min in 1 mM Pi prior to uptake measurement (Supplementary Fig. 4). The C-terminal fragment induced a significant inhibition of *fl*Slc34-mediated Pi uptake even 3 to 5 days after injection. Other constructs, the SPX or the core domain alone, as well as the SPX-core construct, did not show a significant effect on Pi uptake. The inhibitory effect was found to be specific for Pi transport as arginine uptake by rBat (B(0, +)-Type Amino Acid Transporter-Related Heavy Chain, Slc3A1) was not affected by the two XPR1 fragments SPX-core and C-terminus (Fig. 4C).

To summarize our findings so far, despite considerable effort to detect XPR1-mediated efflux of Pi after expression in *Xenopus* oocytes, we found no evidence of such Pi exporter function. In contrast, the C-terminal domain of the modular XPR1 protein appeared to have regulatory potential to reduce *fl*Slc34-mediated Pi uptake.

**Fig. 5:**
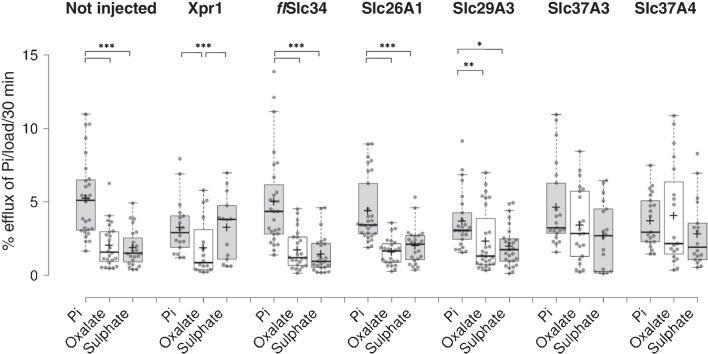
Efflux measurements with oocytes expressing potential Pi exporters. Anion exchangers were identified as potential Pi exporters based on substrate specificity and expression pattern. The transporters (Slc26A1, Slc29A3, Slc37A3 and Slc37A4) were expressed in oocytes, and efflux was assessed in the presence of 2 mM Pi, 50 µM oxalate or 2 mM suphate. Whereas the exogenous exchangers did not show enhanced export activity, though intrinsic export shows preference of Pi over oxalate and sulphate. One-way ANOVA with Tukey’s post hoc test, *p < 0.05, **p < 0.01, ***p < 0.001

There is a finite number of solute transporters that, according to our current knowledge, could potentially mediate cellular Pi efflux, i.e. are widely expressed, localize to the basolateral membrane in epithelial cells and may accept anions as a substrate. According to these three criteria, we identified the transporters Slc26A1, Slc29A3, Slc37A3 and Slc37A4, amplified the cDNA from *Xenopus* kidney RNA and expressed the proteins together with *fl*Slc34 in oocytes. As counter/co-substrate, we included 2 mM Pi, 50 µM oxalate or 2 mM sulphate in the efflux experiments. Overall, the results were inconclusive. In some experiments, Pi uptake into oocytes appeared to be enhanced by approximately twofold; however, this observation concerned different constructs in different experiments. Similar observations were made with efflux experiments where, again, no consistent stimulation could be assigned to a specific construct. The only consistent trend was the enhanced P export in the presence of 2 mM Pi as compared to oxalate and sulphate (Fig. 5).

## Discussion

We explored various avenues to functionally express the candidate Pi export protein XPR1 in *Xenopus* oocytes; the *Xenopus* XPR1 isoform was studied to avoid species incompatibility. The protein was expressed at the oocyte membrane but failed to enhance Pi efflux. Despite co-injection of cRNA encoding potential co-factors and the addition of co-substrates to the efflux medium, no transport activity was associated with XPR1 expression. Unexpectedly, fragments of XPR1 downregulated the co-expressed Pi uptake transporter *fl*Slc34.

Since Giovannini and coworkers put forward XPR1 as the principal Pi exporter in metazoans, a significant number of studies have corroborated its pivotal role in cellular Pi homeostasis. The impact is well documented in the context of familial brain calcification where mutations in XPR1 as well as in SLC20A2, MYORG, PDGFB (platelet-derived growth factor subunit B) and a few other genes lead to mineralization in the basal ganglia and potentially other brain regions (cerebellum, subcortical white matter and thalamus). The presence of bone matrix proteins in affected areas indicates an osteogenic environment [31] where disturbance of the local Pi balance may exacerbate pathological calcification, rather than mutations in SLC20A2 or XPR1, respectively, causing toxic Ca/Pi precipitates [8]. Accordingly, mutations in SLC20A2 or XPR1 have a lower penetrance and a later onset than other mutations associated with familial brain calcification (MYORG, PDGFB and PDGFRB) [2]. Remarkably, the disease-causing mutations within the XPR gene are all in hydrophilic domains, four in the N-terminal SPX domain, one in an extracellular loop and two in the intracellular C-terminus (https://www.uniprot.org/uniprotkb/Q9UBH6/entry). To compare, the mutations that affect transport activity in SLC20A2 are most predominantly localized in transmembrane domains constituting the functional core of the protein [27]. This argument concurs with our findings that XPR1 regulates rather than exports Pi with the SPX domain and C-terminus exhibiting essential functions.

XPR1 is highly expressed in the kidney, especially in proximal tubular cells (https://www.proteinatlas.org/ENSG00000143324-XPR1/single+cell+type/kidney) in line with their pivotal role in Pi reabsorption and whole-body Pi homeostasis. The Na-dependent Pi transport systems SLC34A1 and A3 are expressed in proximal tubular cells and accumulate Pi from the primary urine into the cytosol. Transcellular transport then requires the exit of Pi at the basolateral side of tubular cells, and the high expression of XPR1 implies an involvement in Pi export. Kidney-specific knockout of XPR1 in mice causes Fanconi syndrome and hypophosphatemic rickets [1], underpinning an essential role of XPR1 in transcellular Pi transport. Conversely, patients with mutations in XPR1 show brain calcification but have normal Ca^2+^ and Pi plasma levels [4] suggesting that renal proximal tubular cells are not severely affected. However, the fact that mutations in XPR1 damage cells exposed to a balanced internal milieu and cause a severe brain phenotype without similarly affecting renal tubular cells that show high transcellular Pi transport is counterintuitive. Other means of Pi export may therefore exist.

The experiments interrogating Pi export are challenging. The particular XPR1 isoform as well as the cell model system used for the assay including specific cell culture conditions, i.e. confluency of the cell layer, the use of permeable support, cell differentiation or media composition may all have a decisive influence on Pi transport [26]. Importantly, the often very high levels of Pi used in the experiments may chelate Ca^2+^ ions and affect trans-epithelial resistance and/or cell signalling. As a consequence of these experimental pitfalls, the interpretation of results is challenging, and conclusions may not apply broadly. We have here used *Xenopus laevis* oocytes to assess the functions of XPR1. Oocytes respond to extracellular Pi levels by downregulating intrinsic uptake (Fig. 1D) likely via SLC20A1 and SLC20A2, with regulatory input from XPR1, IP6K1, PPIP5K1, PPIP5K2 and KIDINS220 all of which are expressed at low levels in oocytes [25] (Supplementary Fig. 2). These observations suggest that *Xenopus* oocytes have a functional Pi-sensing system that regulates Pi uptake—and potentially Pi efflux. Accordingly, exogenous *Xenopus* XPR1 is expressed at the cell membrane and could potentially mediate Pi export; however, we found no evidence of such transport activity. Conversely, we found the regulatory activity of XPR1 fragments affecting Pi uptake mediated by *fl*Slc34. Our results (Figs. 3 and 4) support a hypothesis that XPR1 acts as a Pi sensor and regulator of Pi uptake into cells. Accordingly, the different tasks are associated with the three distinct domains, the SPX domain would mediate Pi-sensing, the core domain membrane tethering and membrane localization and the C-terminal part potentially acted as an effector domain to reduce Pi uptake. Under experimental conditions without Pi in Barth’s solution, cytoplasmic Pi would be relatively low and the SPX domain of XPR1 in an inactive state. The addition of Pi to the medium would increase intracellular Pi, stimulate the formation of IP7/8 and lead to downregulation of Pi uptake. Experimental truncation of the SPX domain from XPR1 would release the inhibition and allow slow lateral diffusion of the membrane-tethered core-C-terminus construct to interact with Slc20/Slc34 transporters and downregulate the Pi transport. Conversely, expression kinetics of the C-terminal effector domain and quick diffusion within the cytoplasm make this low molecular weight protein a much more efficient regulator (as compared to the construct integrated in the membrane). We also attempted to demonstrate similar effects with exogenous Slc20a1 and Slc20a2 from *Xenopus*, though results were inconclusive, probably because of the low expression levels of the exogenous transporters.

To conclude, our results contradict the current view of the function of XPR1 as the main Pi exporter. The expression of XPR1 and fragments in *Xenopus* oocytes suggests regulatory, rather than Pi export activity. Unfortunately, we were unable to identify a functional Pi exporter, and the question remains whether this task is mediated by a specific Pi exchanger (Fig. 5 and ref [14]) or, alternatively, whether Pi is traded for another (anionic?) substrate utilizing its electrochemical driving force.

### Supplementary Information

Below is the link to the electronic supplementary material.

Supplementary file1 (XLSX 15 KB)

Supplementary file2 (DOCX 311 KB)

## Data Availability

No datasets were generated or analysed during the current study.

## References

[1] Ansermet C, Moor MB, Centeno G, Auberson M, Hu DZ, Baron R, Nikolaeva S, Haenzi B, Katanaeva N, Gautschi I, Katanaev V, Rotman S, Koesters R, Schild L, Pradervand S, Bonny O, Firsov D (2017). Renal fanconi syndrome and hypophosphatemic rickets in the absence of xenotropic and polytropic retroviral receptor in the nephron. J Am Soc Nephrol.

[2] Balck A, Schaake S, Kuhnke NS, Domingo A, Madoev H, Margolesky J, Dobricic V, Alvarez-Fischer D, Laabs B-H, Kasten M, Luo W, Nicolas G, Marras C, Lohmann K, Klein C, Westenberger A (2021). Genotype–phenotype relations in primary familial brain calcification: systematic MDSGene review. Mov Disord.

[3] Beck L, Leroy C, Beck-Cormier S, Forand A, Salaün C, Paris N, Bernier A, Ureña-Torres P, Prié D, Ollero M, Coulombel L, Friedlander G (2010). The phosphate transporter PiT1 (Slc20a1) revealed as a new essential gene for mouse liver development. PLoS ONE.

[4] Boller F, Boller M, Gilbert J (1977). Familial idiopathic cerebral calcifications. J Neurol Neurosurg Psychiatry.

[5] Bon N, Couasnay G, Bourgine A, Sourice S, Beck-Cormier S, Guicheux J, Beck L (2018). Phosphate (P(i))-regulated heterodimerization of the high-affinity sodium-dependent P(i) transporters PiT1/Slc20a1 and PiT2/Slc20a2 underlies extracellular P(i) sensing independently of P(i) uptake. J Biol Chem.

[6] Bondeson DP, Paolella BR, Asfaw A, Rothberg MV, Skipper TA, Langan C, Mesa G, Gonzalez A, Surface LE, Ito K, Kazachkova M, Colgan WN, Warren A, Dempster JM, Krill-Burger JM, Ericsson M, Tang AA, Fung I, Chambers ES, Abdusamad M, Dumont N, Doench JG, Piccioni F, Root DE, Boehm J, Hahn WC, Mannstadt M, McFarland JM, Vazquez F, Golub TR (2022). Phosphate dysregulation via the XPR1–KIDINS220 protein complex is a therapeutic vulnerability in ovarian cancer. Nature Cancer.

[7] Chande S, Caballero D, Ho BB, Fetene J, Serna J, Pesta D, Nasiri A, Jurczak M, Chavkin NW, Hernando N, Giachelli CM, Wagner CA, Zeiss C, Shulman GI, Bergwitz C (2020). Slc20a1/Pit1 and Slc20a2/Pit2 are essential for normal skeletal myofiber function and survival. Sci Rep.

[8] Chen S-Y, Ho C-J, Lu Y-T, Lin C-H, Lan M-Y, Tsai M-H (2023). The genetics of primary familial brain calcification: a literature review. Int J Mol Sci.

[9] Forster IC, Wagner CA, Busch AE, Lang F, Biber J, Hernando N, Murer H, Werner A (1997). Electrophysiological characterization of the flounder type II Na+/PiCotransporter (NaPi-5) expressed in Xenopus laevis oocytes. J Membr Biol.

[10] Giovannini D, Touhami J, Charnet P, Sitbon M, Battini JL (2013). Inorganic phosphate export by the retrovirus receptor XPR1 in metazoans. Cell Rep.

[11] Guo X-X, Zou X-H, Wang C, Yao X-P, Su H-Z, Lai L-L, Chen H-T, Lai J-H, Liu Y-B, Chen D-P, Deng Y-C, Lin P, Lin H-S, Hong B-C, Yao Q-Y, Chen X-J, Huang D-Q, Fu H-X, Peng J-D, Niu Y-F, Zhao Y-Y, Zhu X-Q, Lu X-P, Lin H-L, Li Y-K, Liu C-Y, Huang G-B, Wang N, Chen W-J (2019). Spectrum of SLC20A2, PDGFRB, PDGFB, and XPR1 mutations in a large cohort of patients with primary familial brain calcification. Hum Mutat.

[12] Hernando N, Gagnon KB, Lederer ED (2021). Phosphate transport in epithelial and nonepithelial tissue. Physiol Rev.

[13] Hernando N, Myakala K, Simona F, Knopfel T, Thomas L, Murer H, Wagner CA, Biber J (2015). Intestinal depletion of NaPi-IIb/Slc34a2 in Mice: renal and hormonal adaptation. J Bone Miner Res.

[14] Ito M, Haito S, Furumoto M, Uehata Y, Sakurai A, Segawa H, Tatsumi S, Kuwahata M, Miyamoto K-I (2007). Unique uptake and efflux systems of inorganic phosphate in osteoclast-like cells. Am J Physiol Cell Physiol.

[15] Jennings ML (2023) Role of transporters in regulating mammalian intracellular inorganic phosphate. Front Pharmacol 14:1163442 10.3389/fphar.2023.1163442 PMC10097972 37063296

[16] Koike M, Uga M, Shiozaki Y, Miyamoto K-I, Segawa H (2023). Regulation of phosphate transporters and novel regulator of phosphate metabolism. Endocrines.

[17] Lederer E (2023). Understanding renal phosphate handling: unfinished business. Curr Opin Nephrol Hypertens.

[18] Legati A, Giovannini D, Nicolas G, Lopez-Sanchez U, Quintans B, Oliveira JR, Sears RL, Ramos EM, Spiteri E, Sobrido MJ, Carracedo A, Castro-Fernandez C, Cubizolle S, Fogel BL, Goizet C, Jen JC, Kirdlarp S, Lang AE, Miedzybrodzka Z, Mitarnun W, Paucar M, Paulson H, Pariente J, Richard AC, Salins NS, Simpson SA, Striano P, Svenningsson P, Tison F, Unni VK, Vanakker O, Wessels MW, Wetchaphanphesat S, Yang M, Boller F, Campion D, Hannequin D, Sitbon M, Geschwind DH, Battini JL, Coppola G (2015). Mutations in XPR1 cause primary familial brain calcification associated with altered phosphate export. Nat Genet.

[19] Levi M, Gratton E, Forster IC, Hernando N, Wagner CA, Biber J, Sorribas V, Murer H (2019). Mechanisms of phosphate transport. Nat Rev Nephrol.

[20] Markovich D (2008). Expression cloning and radiotracer uptakes in Xenopus laevis oocytes. Nat Protoc.

[21] Marks J (2019). The role of SLC34A2 in intestinal phosphate absorption and phosphate homeostasis. Pflugers Arch.

[22] Marks J, Debnam ES, Unwin RJ (2013). The role of the gastrointestinal tract in phosphate homeostasis in health and chronic kidney disease. Curr Opin Nephrol Hypertens.

[23] Meireles AM, Shiau CE, Guenther CA, Sidik H, Kingsley DM, Talbot WS (2014). The phosphate exporter xpr1b is required for differentiation of tissue-resident macrophages. Cell Rep.

[24] Onaga C, Tamori S, Motomura H, Ozaki A, Matsuda C, Matsuoka I, Fujita T, Nozaki Y, Hara Y, Kawano Y, Harada Y, Sato T, Mano Y, Sato K, Akimoto K (2021). High SLC20A1 expression is associated with poor prognoses in claudin-low and basal-like breast cancers. Anticancer Res.

[25] Peshkin L, Lukyanov A, Kalocsay M, Gage RM, Wang D, Pells TJ, Karimi K, Vize PD, Wühr M, and Kirschner MW (2019) The protein repertoire in early vertebrate embryogenesis. bioRxiv 571174

[26] Wagner CA, Rubio-Aliaga I, Hernando N (2019). Renal phosphate handling and inherited disorders of phosphate reabsorption: an update. Pediatr Nephrol.

[27] Wang C, Li Y, Shi L, Ren J, Patti M, Wang T, de Oliveira JRM, Sobrido M-J, Quintáns B, Baquero M, Cui X, Zhang X-Y, Wang L, Xu H, Wang J, Yao J, Dai X, Liu J, Zhang L, Ma H, Gao Y, Ma X, Feng S, Liu M, Wang QK, Forster IC, Zhang X, Liu J-Y (2012). Mutations in SLC20A2 link familial idiopathic basal ganglia calcification with phosphate homeostasis. Nat Genet.

[28] Wege S, Poirier Y (2014). Expression of the mammalian xenotropic polytropic virus receptor 1 (XPR1) in tobacco leaves leads to phosphate export. FEBS Lett.

[29] Werner A, Biber J, Forgo J, Palacin M, Murer H (1990). Expression of renal transport systems for inorganic phosphate and sulfate in Xenopus laevis oocytes. J Biol Chem.

[30] Werner A, Murer H, Kinne RK (1994). Cloning and expression of a renal Na-Pi cotransport system from flounder. Am J Physiol.

[31] Zarb Y, Weber-Stadlbauer U, Kirschenbaum D, Kindler DR, Richetto J, Keller D, Rademakers R, Dickson DW, Pasch A, Byzova T, Nahar K, Voigt FF, Helmchen F, Boss A, Aguzzi A, Klohs J, Keller A (2019). Ossified blood vessels in primary familial brain calcification elicit a neurotoxic astrocyte response. Brain.

[32] Zhou W, Simic P, Zhou IY, Caravan P, Vela Parada X, Wen D, Washington OL, Shvedova M, Pierce KA, Clish CB, Mannstadt M, Kobayashi T, Wein MN, Jüppner H, and Rhee EP (2023) Kidney glycolysis serves as a mammalian phosphate sensor that maintains phosphate homeostasis. J Clin Investig 133 10.1172/JCI164610 PMC10104895 36821389

