# Supplementary material for: XPR1: a regulator of cellular phosphate homeostasis rather than a Pi exporter
Source: Pflugers Arch. 2024 Mar 20;476(5):861–9. doi: 10.1007/s00424-024-02941-0 (PMC11033234; doi:10.1007/s00424-024-02941-0)
Supplement: Supplementary file 2 — Supplementary file2 (DOCX 311 KB) [file 424_2024_2941_MOESM2_ESM.docx]

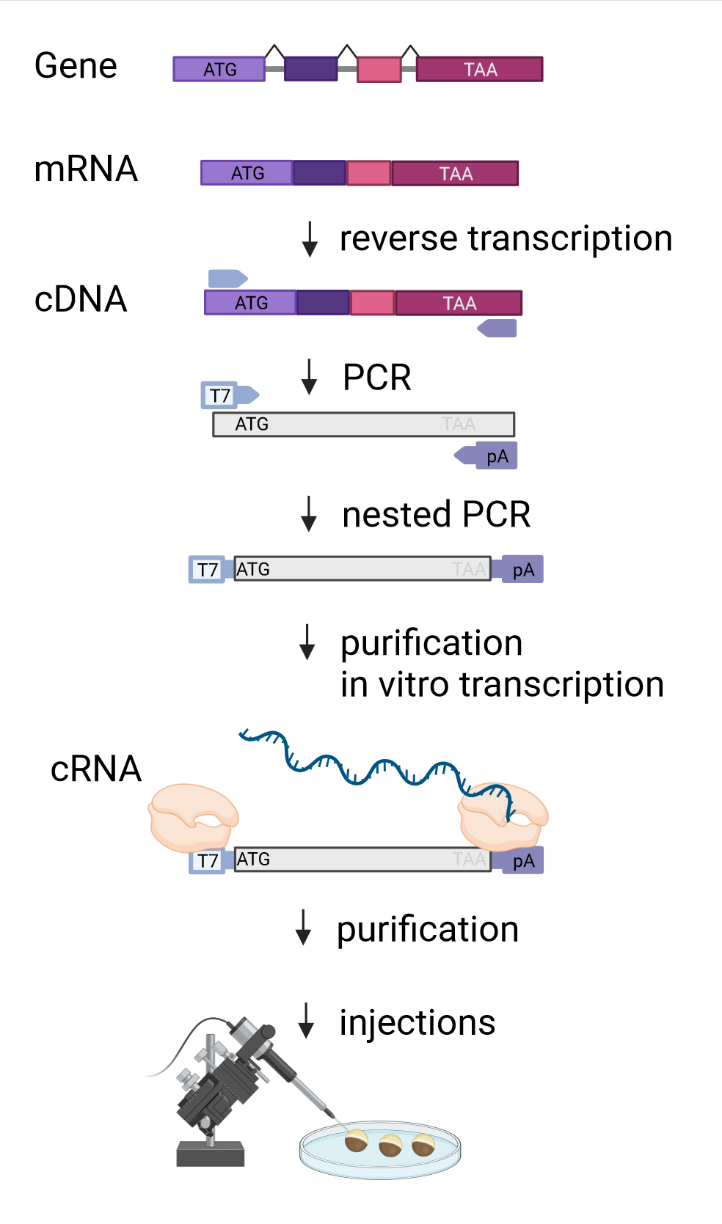


**Supplementary Figure 1:** Strategy to synthesize cRNA from tissue extracted mRNA.

**Supplementary Figure 2:** Expression levels of proteins expressed in stage VI Xenopus oocytes that are potentially involved in sensing and uptake of Pi. Data from <https://www.xenbase.org/xenbase/gene/geneExpressionChart.do?method=drawProtein>.


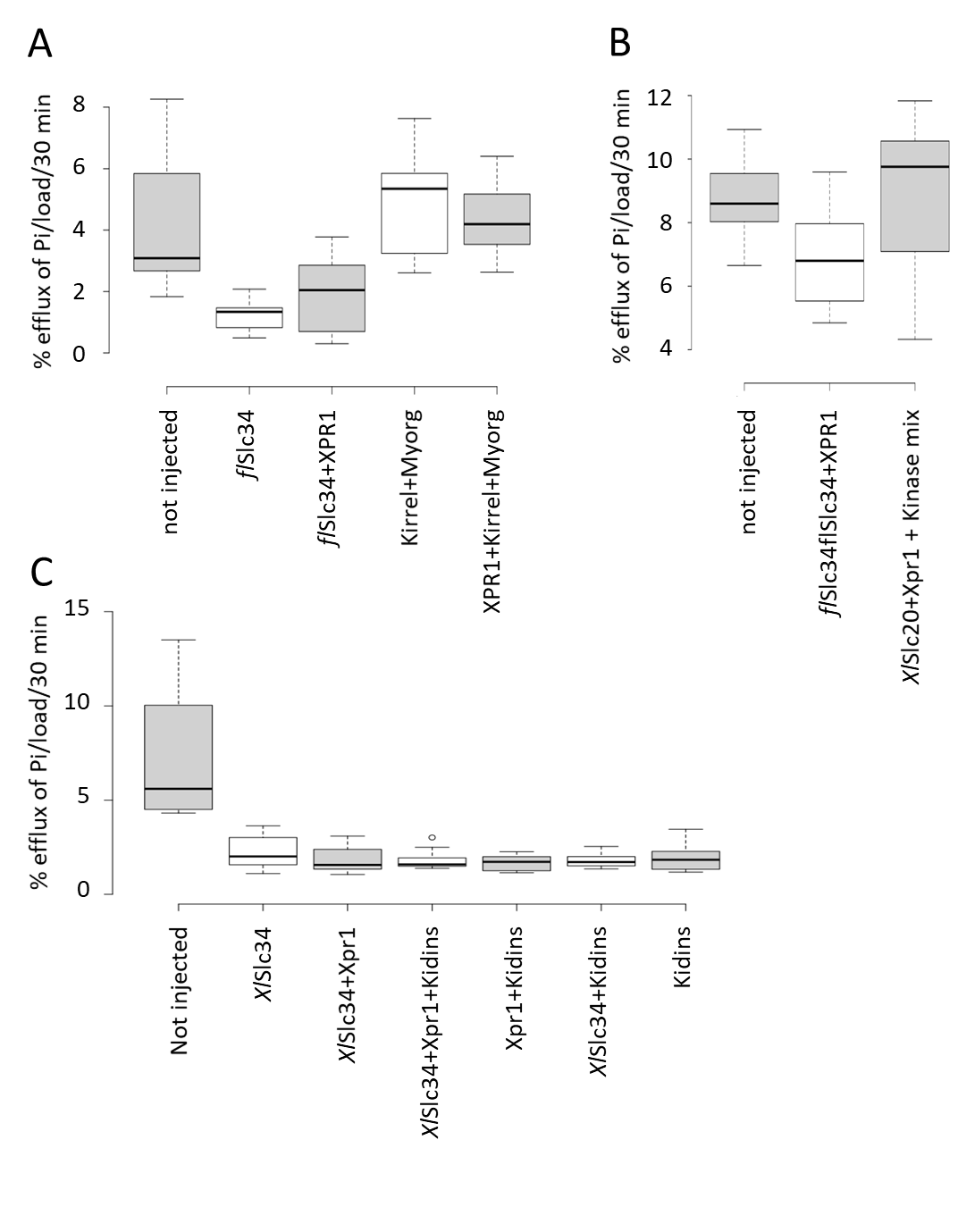


**Supplementary Figure 3:** Efflux experiments testing different co-factors that potentially stimulate the action of Xpr1. A positive effect of the potential Xpr1 modulators would lead to enhanced Pi export through stimulation of endogenously expressed proteins. Addition of exogenous components such as Slc20, Slc34 or Xpr1 would enhance the signal. Though, stimulation of efflux was not observed. (A) Myorg and Kirrel were tested together but failed to enhance Pi export. (B) A mix of cRNA encoding kinases (IP6K1/2, PPIP5K1/2) was injected. (C) cRNA encoding the Xpr1 cofactor Kidins220 was injected in various combinations with XlSlc34 and Xpr1 and had no effect on Pi export. Experiments were performed twice. Center lines show the medians; box limits indicate the 25th and 75th percentiles; whiskers extend 1.5 times the interquartile range from the 25th and 75th percentiles, an outliers is represented by a dot.


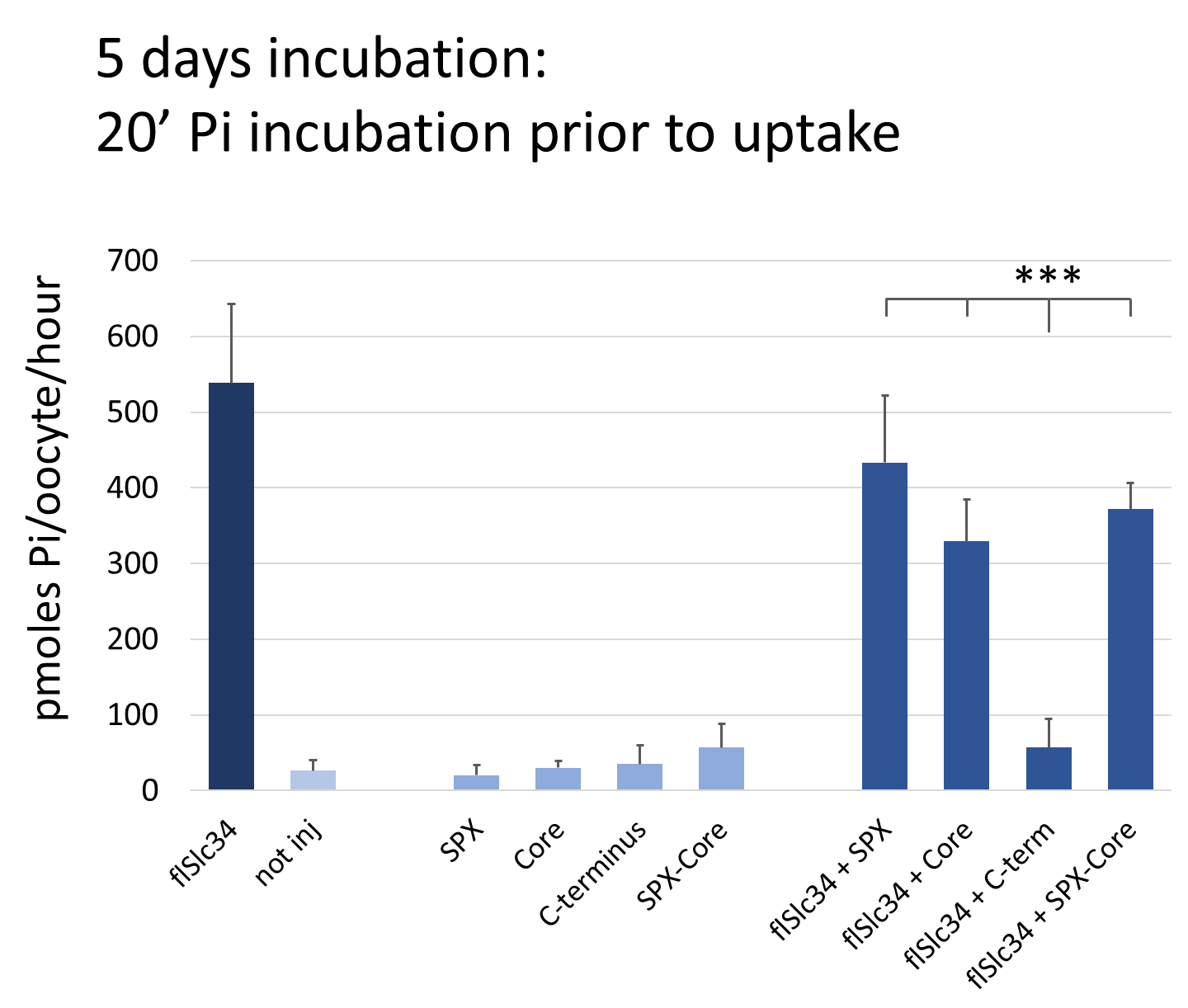


**Supplementary Figure 4:** Pi uptake into oocytes expressing XPR1 protein domains. Cells were injected with constructs of XPR1 domains; SPX- domain, core domain, the C- terminus and XPR1 without C- terminus (SPX-core) and assayed after 5 days. In addition, oocytes were incubated in 1 mM Pi for 20’ prior to the uptake. The pre-incubation reduced the variability between individual oocytes, all groups were similarly affected. (This figure compares to Figure 5B in the main text.) One-Way ANOVA with Tukey’s post hoc test, *** = p<0.001.
